# Meta-analysis of the association between SCARB1 polymorphism and fasting blood lipid levels

**DOI:** 10.18632/oncotarget.20867

**Published:** 2017-09-14

**Authors:** Li-Fang Ye, Ya-Ru Zheng, Qing-Gang Zhang, Jian-Wu Yu, Li-Hong Wang

**Affiliations:** ^1^ Department of Cardiovascular Sciences, Zhejiang Provincial People's Hospital, Hangzhou, Zhejiang, China

**Keywords:** scavenger receptor class B type 1, polymorphism, lipid, sex

## Abstract

Studies have shown that the scavenger receptor class B type 1 (SCARB1) rs5888 polymorphism impacts fasting blood lipid levels differently in men and women. A meta-analysis and statistical tests was therefore performed to determine the relationship between the rs5888 polymorphism and lipid levels in men and women. Twelve studies with 12,147 subjects were selected for this study. In a dominant model, the CT+TT genotype group had lower triglyceride levels than the CC group in men (standardized mean difference (SMD): −0.11; 95% confidence interval (CI): −0.21 to −0.02; *P* = 0.016; I^2^ = 51.5%). No statistical differences were detected in women. Subgroup analysis of different racial groups revealed significant correlation between the SCARB1 rs5888 polymorphism and higher high-density lipoprotein cholesterol (HDL-C) levels (SMD: 0.15; 95% CI: 0.08 to 0.21; *P* ≤ 0.001; I^2^ = 0%) and lower triglyceride levels (SMD: −0.16; 95% CI: −0.26 to −0.04; *P* = 0.013; I^2^ = 60.6%) in non-Asian men. No evidence of heterogeneity was observed when eliminating outlier studies, and no publication bias was detected. This meta-analysis suggests the SCARB1 rs5888 polymorphism is associated with higher HDL-C and lower triglyceride levels in non-Asian men.

## INTRODUCTION

Cardiovascular disease (CVD) is the leading cause of death in developed and many developing countries [1]. Risk factors such as hypertension, dyslipidemia, and adiposity may contribute to the development of CVD. Dyslipidemia is characterized by abnormal blood lipid levels, such as increased low-density lipoprotein cholesterol (LDL-C), triglycerides (TG), and total cholesterol (TC) and decreased high-density lipoprotein cholesterol (HDL-C) [2]. Dyslipidemia is a risk factor for the development of CVD and an important topic of research [2]. Recently, chromosomal regions and specific genes such as cholesterol ester transfer protein (CETP) and scavenger receptor class B type 1 (SCARB1) have been proposed to be associated with blood lipid levels [3]. Thus, analysis of nucleotide polymorphisms is a useful tool to better understand differences in lipid levels.

SCARB1 is an HDL receptor closely coupled with HDL-C that promotes HDL-C uptake and reverse translation [4]. Studies have shown that SCARB1 facilitates lipoprotein metabolism and decreases atherosclerosis in mice. In various animal models, overexpression of SCARB1 reduced blood lipids and ameliorated atherosclerosis [5]. In contrast, higher blood lipid levels and occurrence of atherosclerosis were detected in SCARB1−/− mice [6]. In LDL receptor–deficient mice, attenuated expression of SCARB1 was associated with increased LDL-C and accelerated atherosclerosis [7, 8]. The association between SCARB1 polymorphism and fasting blood lipid levels has been reported in diverse populations [9–20]. Early studies by Acton et al. [11] identified a single nucleotide polymorphism (SNP) in the SCARB1 gene, called rs5888, that contains a C-to-T substitution at the 1050 base pair cDNA position of exon 8. Interestingly, studies have shown that rs5888 has a different effect on fasting blood lipid levels in men and women [10–12, 14, 18, 20]. SCARB1 CC genotype carriers had significantly lower levels of LDL-C than the TT genotype group and higher levels of TG compared with the CT genotype group. In addition, no association between the SCARB1 polymorphism and dyslipidemia was found in women [9]. In contrast, Morabia et al. revealed that female T carriers had higher LDL-C and male T carriers had lower HDL-C [17]. Moreover, another study reported that, in men age 65 to 74 years, those with the TT genotype had higher HDL-C levels and a lower risk of myocardial infarction than those with the CC genotype [18]. Sex-specific effects on blood lipids were also controversial in other studies [14, 21, 22]. The mechanism by which SCARB1 rs5888 regulates blood lipid levels remains to be determined. As a result of the inconsistency of existing research, we performed a systematic meta-analysis to investigate the association between SCARB1 polymorphism and fasting blood lipid levels.

## MATERIALS AND METHODS

### Literature search strategy

We searched for cross-sectional and case-control studies from January 1990 to June 2015 in PubMed, Embase, China National Knowledge Infrastructure, Wanfang Data, and China Biology Medicine databases. The keywords used for this search were as follows: “scavenger receptor AND (genetic OR variant OR variation OR mutation OR mutated OR polymorphism OR snp) AND [(low-density lipoprotein cholesterol) OR (high-density lipoprotein cholesterol) OR LDL-C OR HDL-C or triglyceride or (total cholesterol)].” The reference lists of the included studies were also searched for relevant results.

### Literature selection criteria

**Figure 1:**
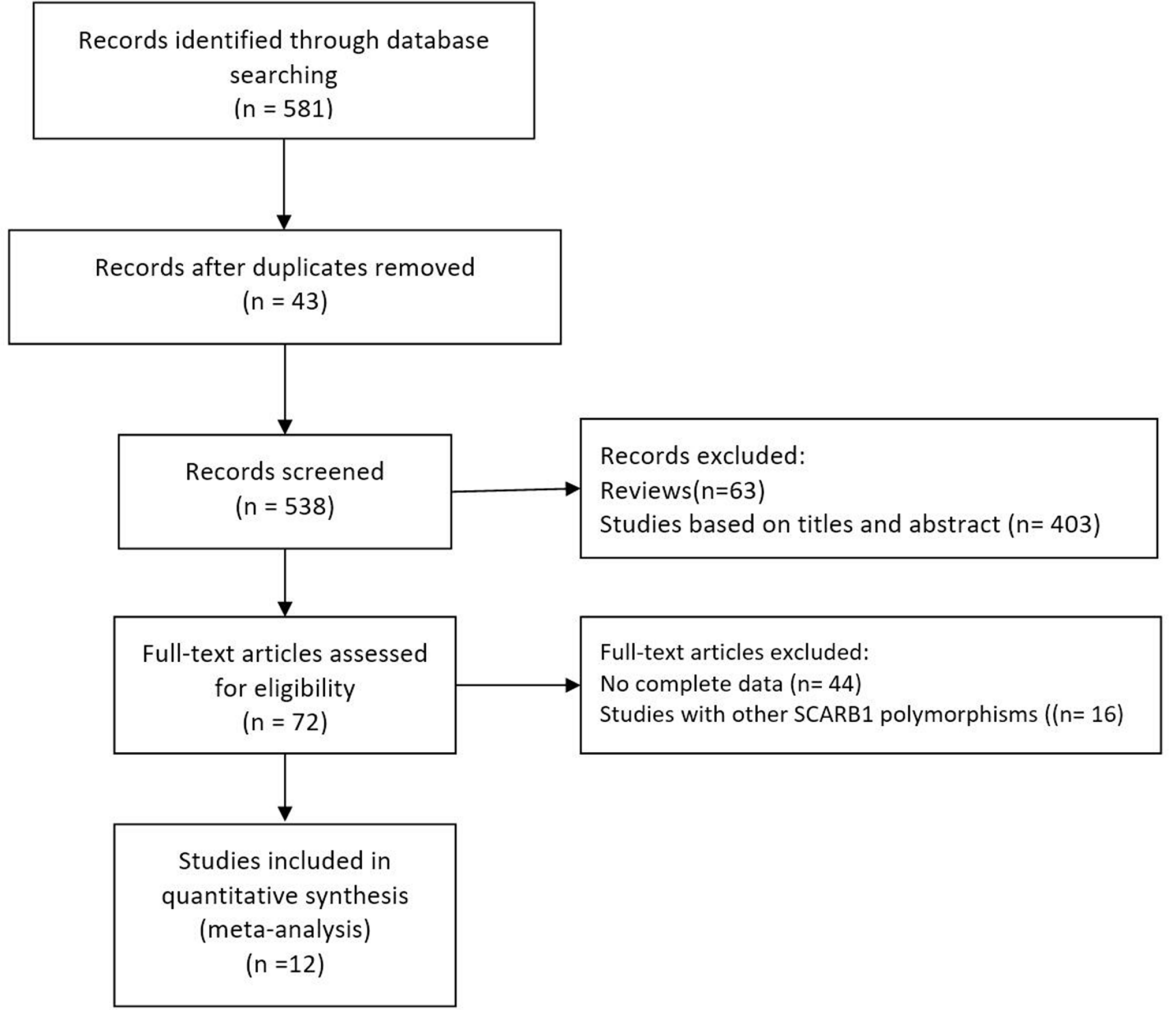
Flow diagram of the study selection procedure used for this meta-analysis of SCARB1 rs5888 polymorphism and fasting blood lipid levels

Two investigators (Li-fang Ye and Ya-ru Zheng) independently reviewed all the studies retrieved from the database searches. We followed guidelines from the Preferred Reporting Items for Systematic Reviews and Meta-analyses (PRISMA) statement (Figure 1 and Supplementary Table 1). The selection criteria for this meta-analysis were as follows: (1) studied the relationship between SCARB1 polymorphisms and fasting blood lipid levels; (2) measured at least one of the lipid phenotypes (TC, HDL-C, LDL-C, or TG); and (3) provided data for men and women separately. Each group (case and control groups) was treated as one single study, and genotype frequency in control populations was tested for Hardy-Weinberg equilibrium. We excluded studies when the genotype frequency was not possible to extract from either the published results or by contacting the authors. If multiple reports presented the same data, the more detailed study was chosen for further analysis.

### Data extraction

Two reviewers (Li-fang Ye and Ya-ru Zheng) extracted the papers independently. We initially reviewed the article titles and abstracts and then excluded those that did not fit the inclusion criteria. All the included studies were extensively reviewed. Any disagreements were resolved by discussion. For each included study, we assessed methodologic rigor and quality using STREGA (Strengthening the Reporting of Genetic Association Studies) guidelines, an extension of STROBE (Strengthening the Reporting of Observational Studies in Epidemiology) guidelines specifically developed to assess quality of genetic association studies. The information that was extracted included first author name, year of publication, ethnicity, sample size, sex, age, health condition, genotype information (genotyping method, number of genotypes), and relationship between genotypes and fasting blood lipid levels.

### Statistical analysis

All data were expressed as mean ± standard deviation. All analyses were performed using the STATA software package v12.0 (Stata Corporation, College Station, TX). We used a dominant model (CT + TT vs CC) as a result of the low rate of the TT genotype in the Asian population. When one article included more than one subpopulation (e.g., female and male subjects, coronary heart disease and normal population, subjects with different ethnicity), the subpopulation was analyzed as a separate comparison. In addition, we divided the ethnic subgroups into Asian and non-Asian. The meta-analyses on the subgroups were performed with at least four comparisons to ensure adequate statistical power.

The differences in the variables between the genotypes were expressed as the pooled standardized mean difference (SMD) with 95% confidence interval (CI), and the random effects model was used for all analyses [23]. We assessed heterogeneity using χ^2^-based Q-tests. I^2^6 values of 25%, 50%, and 75% represent low, medium, and high levels of heterogeneity, respectively. Potential heterogeneity was detected using the Galbraith plot, and the pooled SMD was recalculated with the outlier studies removed. Publication bias was assessed using the Begg's funnel plot and the Egger's linear regression test. *P* values less than 0.05 were considered statistically significant.

## RESULTS

### Characteristics of the included studies

Initial search of the literature yielded 581 publications. Forty-three studies had subject overlap with other publications. Sixty-three publications were reviews. Four hundred three studies were excluded based on their title and abstract. Next, full-text articles were retrieved and assessed based on inclusion criteria. Overall, 60 articles were excluded for the following reasons: 44 articles did not provide complete data for this meta-analysis, and 16 articles presented data on other polymorphisms. Finally, 12 studies [9–14, 16–20, 24] were selected for the meta-analysis. The flow diagram of the selection process of eligible studies is presented in Figure 1.

The characteristics of the 12 included studies are summarized in Table 1. Individual articles were assessed for quality based on STREGA guidelines (Supplementary Table 2). The patients in each eligible study were divided into subgroups based on sex (male and female). The male SCARB1 rs5888 subgroup (Table 2) included nine studies and 11 separate comparisons of the levels of LDL-C, TC, and TG [9, 11, 12, 14, 17–20, 24] and 10 studies and 12 separate comparisons of the levels of HDL-C [9, 11–14, 17–20, 24]. The female SCARB1 rs5888 subgroup (Table 3) included eight studies and 10 separate comparisons of the levels of LDL-C and TC [9, 11, 12, 14, 17, 18, 20, 24], nine studies and 11 separate comparisons of the levels of TG [9–12, 14, 17, 18, 20, 24], and 10 studies and 14 separate comparisons of the levels of HDL-C [9–12, 14, 16–18, 20, 24].

**Table 1:** Characteristics of 12 studies included in the meta-analysis

First author	Year	Country	Age	Sample size	Gender	Study population	Outcomes	Ethnicity
**Acton**	**1999**	Spain	37.23 ± 13.38	489	M/F	healthy subjects	TG, TC, HDL-C, LDL-C	non-Asian
**Osgood**	**2003**	America	51.7	2630	M/F	randomly selected subjects	TG, TC, HDL-C, LDL-C	non-Asian
**Morabia**	**2004**	Switzerland	50.24 ± 10.32	1756	M/F	randomly selected subjects	TG, TC, HDL-C, LDL-C	non-Asian
**Tanaka**	**2007**	Argentina	22.74 ± 4.99	59	M	healthy male	TG, TC, HDL-C, LDL-C	non-Asian
**Wu1**	**2012**	China	40.22 ± 15.18	598	M/F	Bai Ku Yao, randomly selected subjects	TG, TC, HDL-C, LDL-C	Asian
**Wu2**	**2012**	China	52.55 ± 14.78	801	M/F	Mulao, randomly selected subjects	TG, TC, HDL-C, LDL-C	Asian
**Wu3**	**2012**	China	51.40 ± 15.14	807	M/F	Han, randomly selected subjects	TG, TC, HDL-C, LDL-C	Asian
**Smalinskiene**	**2013**	Lithuanian	46.78 ± 10.54	1025	M/F	healthy subjects	TG, TC, HDL-C, LDL-C	non-Asian
**McCarthy1**	**2003**	America	60.9 ± 10.7	304	F	subjects from families originally ascertained for type 2 diabetes from Ashkenazi	HDL-C	non-Asian
**McCarthy2**	**2003**	America	60.4 ± 12.5	328	F	subjects from families originally ascertained for type 2 diabetes from Helsinki	HDL-C	non-Asian
**McCarthy3**	**2003**	America	59.9 ± 12.5	215	F	subjects from families originally ascertained for type 2 diabetes from Malmo	HDL-C	non-Asian
**McCarthy4**	**2003**	America	47.6	100	F	premature CAD subjects	HDL-C, TG	non-Asian
**Stanislovaitiene**	**2013**	Lithuania	Non	1976	M/F	randomly selected subjects	TG, TC, HDL-C, LDL-C	non-Asian
**Boekholdt**	**2006**	Netherlands	56 ± 8	546	M	CAD subjects	HDL-C	non-Asian
**Guo1**	**2015**	China	55.80 ± 7.72	143	M/F	healthy subjects	TG, TC, HDL-C, LDL-C	Asian
**Guo2**	**2015**	China	60.55 ± 8.42	370	M/F	CAD subjects	TG, TC, HDL-C, LDL-C	Asian

M: male, F: female, CAD: coronary heart disease, TG: triglyceride, TC: total cholesterol, LDL-C: low-density lipoprotein cholesterol, HDL-C: high-density lipoprotein cholesterol

**Table 2:** Fasting blood lipid levels of the studies included in the male SCARB1 rs5888 subgroup

Author/Year	CC									CT*									TT								
	Sample size	TC, mmol/L		LDL-C, mmol/L		HDL-C, mmol/L		TG, mmol/L		Sample size	TC, mmol/L		LDL-C, mmol/L		HDL-C, mmol/L		TG, mmol/L		Sample size	TC, mmol/L		LDL-C, mmol/L		HDL-C, mmol/L		TG, mmol/L	
		MEAN	SD	MEAN	SD	MEAN	SD	MEAN	SD		MEAN	SD	MEAN	SD	MEAN	SD	MEAN	SD		MEAN	SD	MEAN	SD	MEAN	SD	MEAN	SD
Acton.1999	54	5.48	1.14	3.72	1.14	1.16	0.36	1.11	0.66	104	5.28	1.21	3.59	1.21	1.19	0.49	1.08	0.60	34	5.40	1.14	3.67	1.09	1.24	0.39	1.06	0.67
Osgood.2003	327	5.22	1.08	3.41	0.90	1.09	0.36	1.39	0.18	556	5.21	1.18	3.38	1.18	1.12	0.24	1.33	0.24	294	5.10	1.03	3.26	0.86	1.15	0.34	1.31	0.17
Morabia.2004	280	5.76	1.00	3.92	0.84	1.17	0.33	1.26	0.50	436	5.77	1.04	3.90	0.84	1.22	0.21	1.22	0.42	149	5.80	0.98	3.90	0.85	1.24	0.24	1.24	0.49
Tanaka.2007	21	4.10	0.70	2.50	0.60	1.20	0.30	0.90	0.20	25	4.00	0.60	2.40	0.60	1.30	0.30	0.90	0.50	13	3.60	0.60	2.00	0.60	1.20	0.30	0.90	0.40
Wu1.2012	176	4.39	1.09	2.54	0.92	1.70	0.49	1.18	0.95	99	4.43	1.01	2.55	0.86	1.74	0.46	1.05	0.69	11	3.97	1.07	2.35	0.99	1.30	0.45	1.25	0.61
Wu2.2012	191	5.19	1.27	2.93	0.82	1.74	0.39	1.16	0.90	161	5.22	1.60	2.95	0.86	1.76	0.58	1.17	1.36	27	4.99	1.20	2.83	0.87	1.67	0.49	0.94	0.99
Wu3.2012	193	5.09	1.15	2.84	0.78	1.75	0.51	1.14	1.04	140	5.09	0.97	2.99	0.92	1.66	0.35	1.16	1.08	22	5.34	1.09	2.94	0.75	1.46	0.31	1.68	2.61
Smalinskiene.2013	149	5.30	1.10	3.29	0.98	1.26	0.49	1.68	0.40	212	5.29	1.02	3.40	1.02	1.32	0.44	1.53	0.49	63	5.56	1.03	3.66	0.95	1.27	0.40	1.60	0.44
Stanislovaitiene. 2013	307	5.49	1.05	3.48	1.05	1.35	0.35	1.50	0.70	440	5.49	1.05	3.53	1.05	1.38	0.42	1.43	0.84	155	5.70	1.12	3.68	1.00	1.36	0.37	1.57	0.75
Boekholdt.2006	157					0.88	0.19			248					0.94	0.23			141					0.93	0.22		
GUO1.2015	31	4.94	0.98	2.84	0.87	1.18	0.27	2.46	2.60	20	4.94	0.72	3.12	0.55	1.22	0.19	1.50	0.54									
GUO2.2015	139	5.00	0.92	2.93	0.71	1.14	0.25	1.73	1.33	79	4.88	0.75	2.95	0.72	1.12	0.23	1.72	0.78									

*In GUO1 and GUO2, data of “CT” and “TT” phenotype were merged. SD: standard deviation

**Table 3:** Fasting blood lipid levels of the studies included in the female SCARB1 rs5888 subgroup

Author/Year	CC									CT*									TT								
	Sample size	TC, mmol/L		LDL-C, mmol/L		HDL-C, mmol/L		TG, mmol/L		Sample size	TC, mmol/L		LDL-C, mmol/L		HDL-C, mmol/L		TG, mmol/L		Sample size	TC, mmol/L		LDL-C, mmol/L		HDL-C, mmol/L		TG, mmol/L	
		MEAN	SD	MEAN	SD	MEAN	SD	MEAN	SD		MEAN	SD	MEAN	SD	MEAN	SD	MEAN	SD		MEAN	SD	MEAN	SD	MEAN	SD	MEAN	SD
Acton.1999	73	5.33	1.27	3.39	1.09	1.58	0.41	0.76	0.40	148	5.07	1.09	3.05	0.98	1.66	0.44	0.79	0.43	37	4.97	1.09	3.00	0.93	1.66	0.57	0.65	0.25
Osgood.2003	341	5.32	0.92	3.33	0.92	1.44	0.37	1.06	0.18	591	5.25	0.97	3.23	0.97	1.44	0.49	1.08	0.24	307	5.17	1.05	3.16	0.88	1.47	0.35	1.03	0.18
Morabia.2004	248	5.56	0.94	3.58	0.94	1.50	0.31	0.95	0.47	467	5.70	0.86	3.72	0.86	1.51	0.43	0.95	0.43	176	5.80	0.93	3.79	0.93	1.52	0.40	1.00	0.40
Wu1.2012	183	4.22	0.76	2.53	0.58	1.63	0.33	0.90	0.51	120	4.45	0.78	2.72	0.64	1.65	0.35	1.00	0.61	9	4.19	0.84	2.57	0.74	1.51	0.24	1.05	0.38
Wu2.2012	204	4.94	1.07	2.93	0.83	1.78	0.40	1.01	0.67	187	5.01	1.29	3.01	0.99	1.76	0.43	1.04	0.63	31	4.96	1.29	3.01	1.13	1.56	0.44	1.12	0.69
Wu3.2012	252	4.90	1.08	2.86	0.89	1.75	0.42	0.97	0.86	162	4.89	0.87	2.81	0.82	1.85	0.80	1.04	0.74	38	4.72	1.02	2.82	0.79	1.66	0.35	1.00	0.75
Smalinskiene.2013	215	5.31	1.03	3.27	0.88	1.42	0.44	1.49	0.47	287	5.29	1.02	3.23	0.85	1.42	0.34	1.46	0.26	99	5.25	0.99	3.09	0.90	1.49	0.40	1.45	0.36
McCarthy1	61					1.13	0.42			138					1.14	0.37			72					1.27	0.37		
McCarthy2	123					1.23	0.31			157					1.24	0.36			46					1.34	0.29		
McCarthy3	59					1.21	0.33			104					1.32	0.40			46					1.33	0.35		
McCarthy4	30					1.15	0.30	2.59	2.04	47					1.08	0.32	2.84	2.59	23					1.05	0.27	2.31	1.70
Stanislovaitiene. 2013	389	5.63	0.98	3.51	0.98	1.49	0.39	1.42	0.59	503	5.65	1.14	3.52	0.91	1.48	0.45	1.45	0.68	182	5.64	1.05	3.49	0.92	1.48	0.39	1.47	0.66
GUO1.2015	55	4.70	0.86	2.72	0.69	1.36	0.25	1.44	0.85	37	4.90	1.00	3.03	0.76	1.30	0.30	1.48	0.63									
GUO2.2015	100	5.32	1.02	3.25	0.89	1.27	0.26	1.81	1.55	52	5.36	0.93	3.28	0.79	1.29	0.25	1.68	0.87									

*In GUO1 and GUO2, data of “CT” and “TT” phenotype were merged. SD: standard deviation.

### Associations with lipid levels

**Figure 2:**
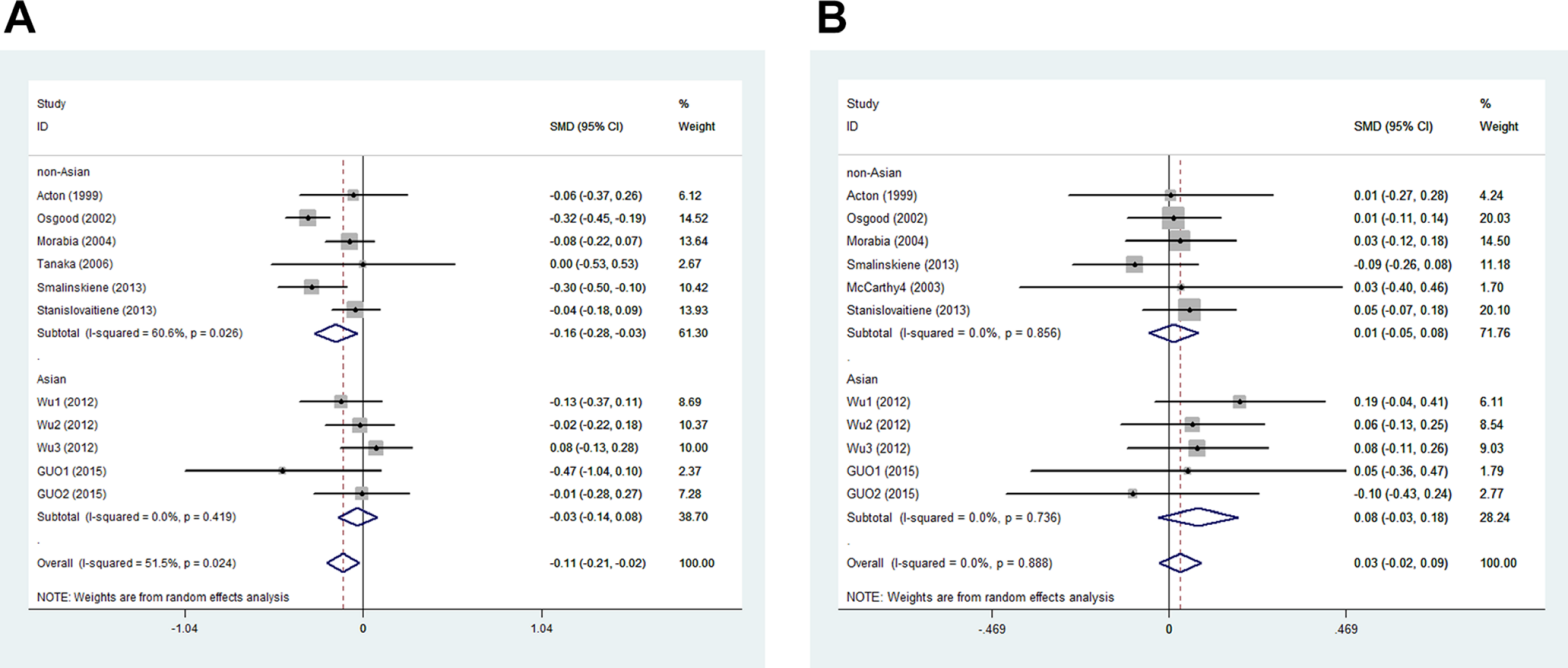
Forest plot of the association between SCARB1 rs5888 polymorphism and triglyceride levels in men (**A**) and women (**B**) (CC vs CT+TT).

**Figure 3:**
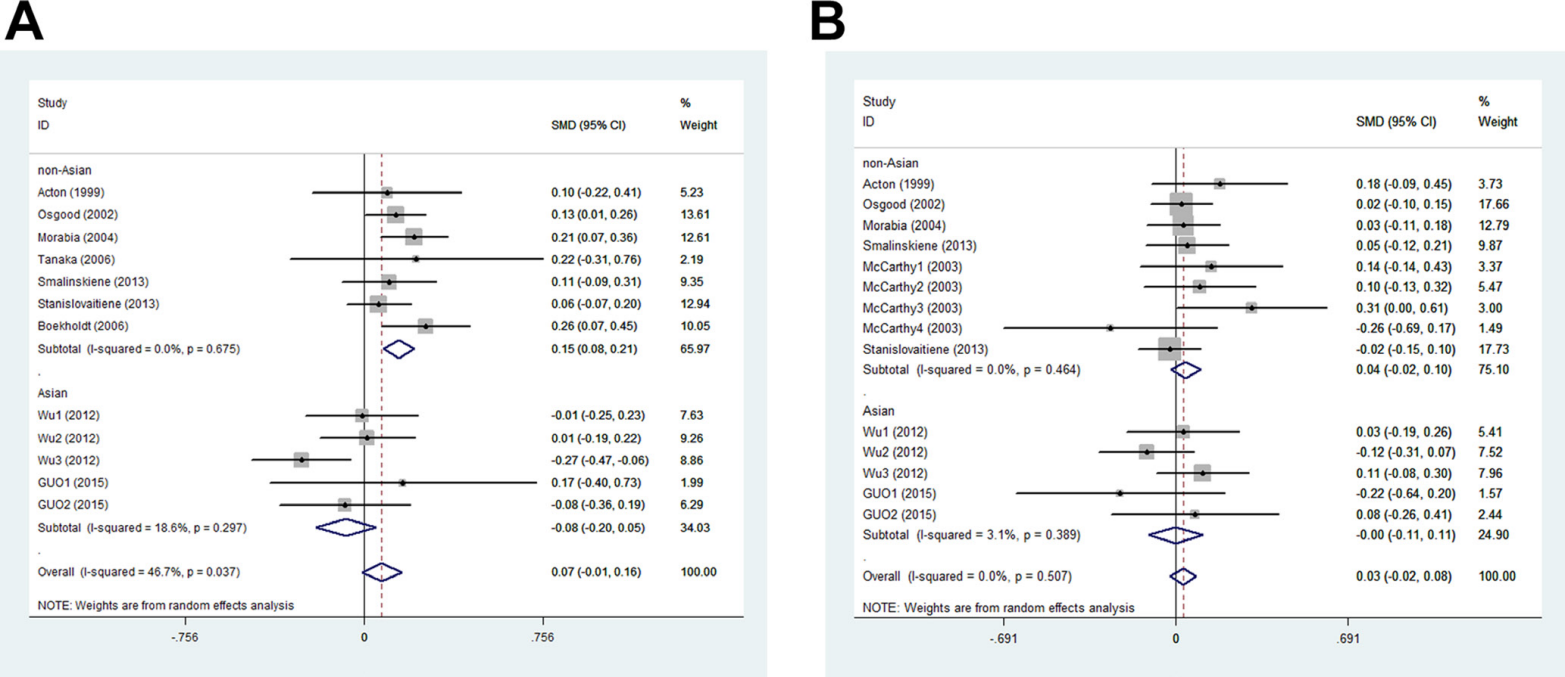
Meta-analysis of the association between SCARB1 rs5888 polymorphism and high-density lipoprotein cholesterol levels in men (**A**) and women (**B**) (CC vs CT+TT).

**Figure 4:**
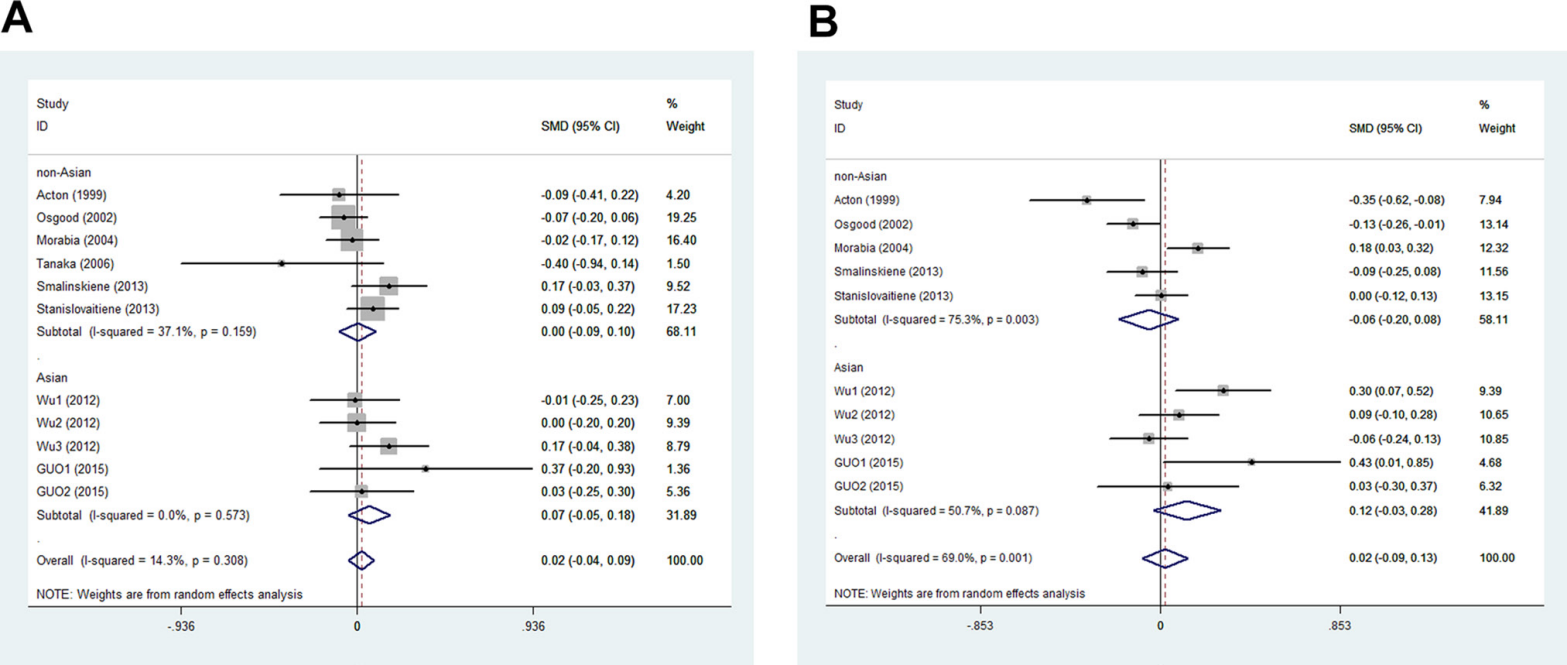
Forest plot of the association between SCARB1 rs5888 polymorphism and low-density lipoprotein cholesterol levels in men (**A**) and women (**B**) (CC vs CT+TT).

**Figure 5:**
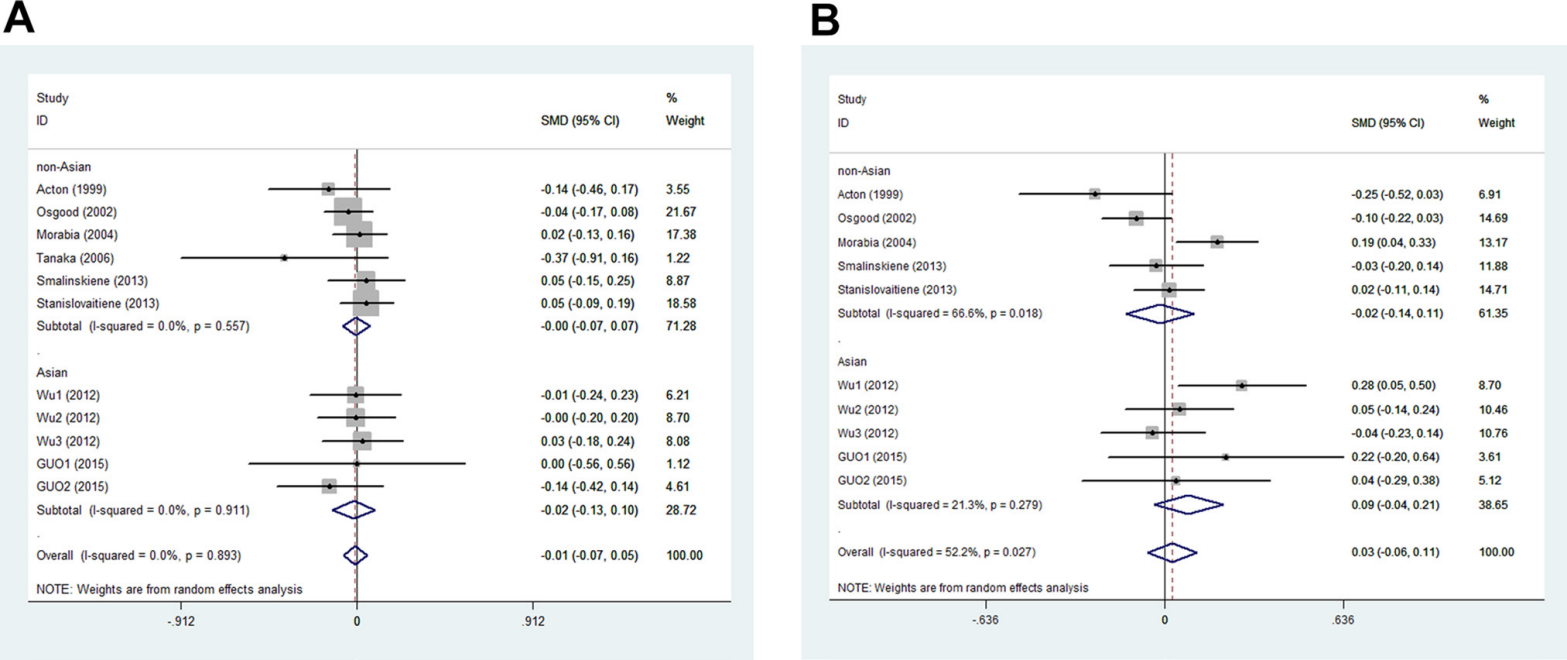
Association between SCARB1 rs5888 polymorphism and total cholesterol levels in men (**A**) and women (**B**) (CC vs CT+TT).

In men, the pooled data for SCARB1 rs5888 indicated that the CT+TT genotype group had lower levels of TG (SMD: −0.11; 95% CI: −0.21 to −0.02; *P* = 0.016; I^2^ = 51.5%) than the CC genotype group (Figure 2A). No statistically significant difference was detected between the CC and CT+TT groups for the levels of HDL-C (SMD: 0.07; 95% CI: −0.01 to 0.16; *P* = 0.081; I^2^ = 46.7%) (Figure 3A), LDL-C (SMD: 0.02; 95% CI: −0.04 to 0.09; *P* = 0.465; I^2^ = 14.3%) (Figure 4A), and TC (SMD: −0.01; 95% CI: −0.07 to 0.05; *P* = 0.83; I^2^ = 0%) (Figure 5A). Subsequently, the subgroups were analyzed based on subjects’ ethnicity. The SCARB1 rs5888 polymorphism was significantly correlated with higher HDL-C levels (SMD: 0.15; 95% CI: 0.08 to 0.21; *P* ≤ 0.001; I^2^ = 0%) (Figure 3A) and lower TG levels (SMD: −0.16; 95% CI: −0.26 to −0.04; *P* = 0.013; I^2^ = 60.6%) in the non-Asian population (Figure 2A). No significant association between SCARB1 rs5888 polymorphism and plasma lipid levels was detected in the Asian population.

In women, the pooled SCARB1 rs5888 data for the comparison between the CC and CT+TT groups demonstrated no statistically significant difference in the levels of HDL-C (SMD: 0.03; 95% CI: −0.02 to 0.08; *P* = 0.248; I^2^ = 0%) (Figure 3B), LDL-C (SMD: 0.02; 95% CI: −0.09 to 0.13; *P* = 0.715; I^2^ = 69.0%) (Figure 4B), TC (SMD: 0.03; 95% CI: −0.06 to 0.11; *P* = 0.548; I^2^ = 52.2%) (Figure 5B), and TG (SMD: 0.03; 95% CI: −0.02 to 0.09; *P* = 0.272; I^2^ = 0%) (Figure 2B). The ethnicity subgroup analyses indicated no significant correlation between the SCARB1 rs5888 polymorphism and plasma lipid levels in the Asian and non-Asian populations.

### Heterogeneity analysis

**Figure 6:**
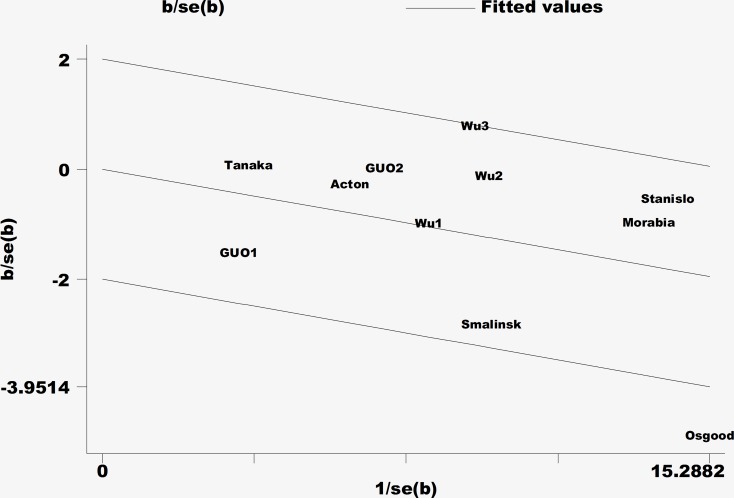
Galbraith plot of the heterogeneity of triglycerides in the male population

The majority of results for the fasting blood lipid levels revealed that the I^2^ values of heterogeneity were less than 50%. Only the levels of heterogeneity of TG in men and LDL-C and TC in women were medium. To identify the studies that contributed to this heterogeneity, a Galbraith plot analysis was performed. The results indicated that a single study [14] was the main contributor for the heterogeneity of TG in men (Figure 6). In women, four studies [11, 14, 17, 20] contributed to the heterogeneity of LDL-C and two [17, 20] to the heterogeneity of TC. The heterogeneity was effectively removed or decreased after exclusion of these outlier studies; however, the SMD values and their 95% CIs did not change significantly (TG in men: SMD: −0.08, 95% CI: −0.14 to −0.01, *P* = 0.035, I^2^ = 4.9%, *P*_heterogeneity_ = 0.396; LDL-C in women: SMD: 0.01, 95% CI: −0.08 to 0.10, *P* = 0.868, I^2^ = 20%, *P*_heterogeneity_ = 0.283; TC in women: SMD: −0.03, 95% CI: −0.10 to 0.03, *P* = 0.326, I^2^ = 0%, *P*_heterogeneity_ = 0.494).

### Publication bias

The Egger's test revealed that there was no publication bias in the analyses for HDL-C (men: t = −0.83, *P* = 0.427; women: t = 0.37, *P* = 0.715), LDL-C (men: t = 0.15, *P* = 0.884; women: t = 0.74, *P* = 0.483), TC (men: t = −1.98, *P* = 0.079; women: t = 0.5, *P* = 0.633), and TG (men: t = 0.44, *P* = 0.669; women: t = 0.03, *P* = 0.974). The shapes of the funnel plots do not show evidence of asymmetry (Supplementary Figures 1 and 2).

## DISCUSSION

Various candidate genes have been reported as predisposing factors for dyslipidemia, including those involved in lipid transport and metabolism [3]. It is well established that SCARB1 facilitates HDL-C clearance through selective uptake of cholesteryl ester from HDL-C to the liver and mediates HDL-C cellular flux of free cholesterol [25, 26]. In addition, SCARB1 aides in the clearance of non-HDL-C particles [27]. Studies have revealed that SCARB1 rs5888 polymorphism is associated with fasting blood levels of TG [19, 20] and LDL-C [11, 14]. Interestingly, many of these studies show different effects of the polymorphisms in men and women, suggesting a possible interaction with the sex hormone signaling network [11, 17, 20, 21].

To our knowledge, we present here the first meta-analysis to investigate the association between SCARB1 polymorphisms, sex, and lipid levels. We included a total of 12 publications including 12,147 subjects. Our results demonstrated a significant correlation between the SCARB1 rs5888 polymorphism and TG levels that was dependent on the presence of the T allele in men. No significant association between rs5888 polymorphism and plasma lipid levels was detected in women. The ethnicity subgroup analyses indicated that the rs5888 polymorphism was significantly correlated with higher HDL-C levels and lower TG levels in non-Asian men but not in Asian men. No significant association between SCARB1 rs5888 polymorphism and plasma lipid levels was identified in either Asian or non-Asian women.

SCARB1 has been identified as a physiologically important HDL receptor; however, it is also a multiligand receptor participating in the metabolism of other plasma lipoproteins [11]. The mechanism of the SCARB1 SNP rs5888 effect on the lipid profile remains undetermined because polymorphisms in this SNP do not lead to a change in the amino acid sequence of the SCARB1 protein [11, 17]. Many factors could have contributed to the sex- and ethnicity-dependent differences found in our study, such as the higher T allele frequency in the non-Asian populations and the influence of sex hormones [12, 20]. Chiba-Falek et al. found that polymorphisms of SCARB1 are associated with HDL-C and TG levels in an endogenous estrogen-dependent fashion [28]. The downregulation of SCARB1 by estrogen has been demonstrated in humans as well [28]. It could account for the underlying sex differences in SCARB1 genetic associations with lipids in this meta-analysis. In addition, environmental factors such as alcohol and tobacco use, lifestyle, and other social characteristics could also have affected our results. Undoubtedly, the relationship between SCARB1 and lipid is complex. Additional studies are required to fully understand the exact genetic basis and to more precisely define the clinical phenotype associated with these genetic variants. Nonetheless, the present investigation identifies genetic variation in SCARB1 as a potentially important determinant of HDL-C and TG levels in non-Asian men at risk for CVD.

High heterogeneity can potentially affect the interpretation of results. In our study, heterogeneity was relatively low, considering that most of the I^2^ values were less than 50%. Medium levels of heterogeneity were observed for TG levels in men and LDL-C and TC levels in women. This heterogeneity could be attributed to the variable genetic and environmental factors influencing the different groups in the included studies. Fasting blood lipid levels and CVD are affected by both genetic factors and environmental factors, such as diet, lifestyle, and physical activity. The heterogeneity was effectively removed or decreased, but the SMD values and their 95% CIs did not change significantly after exclusion of the outlier studies by using a Galbraith plot. This supported the reliability of the results in our meta-analysis.

However, a few limitations of this meta-analysis should be considered. First, the sample size of the included studies was relatively small and might have been insufficient to achieve adequate statistical power for detecting any small effects. A larger sample size is required to further investigate any association between the SCARB1 polymorphism and blood lipid levels. Second, dyslipidemia is also influenced by other genes and environmental factors; however, this was not addressed in this study due to the lack of data from the included studies. A more comprehensive investigation of the interactions of the rs5888 polymorphism with other polymorphic loci or environmental factors and lipid levels is needed.

In conclusion, our analysis demonstrated that the SCARB1 rs5888 polymorphism is associated with higher HDL-C levels and lower TG levels in non-Asian men. Additional studies with larger sample sizes should be conducted to clarify the association between SCARB1 polymorphisms, sex, and lipid levels. Studies investigating the functions of these polymorphisms are also needed.

## SUPPLEMENTARY FIGURES AND TABLES






