# Supplementary material for: Meta-analysis of the association between SCARB1 polymorphism and fasting blood lipid levels
Source: Oncotarget. 2017 Sep 14;8(46):81145–53. doi: 10.18632/oncotarget.20867 (PMC5655269; doi:10.18632/oncotarget.20867)
Supplement: Supplementary file 1 [file oncotarget-08-81145-s001.pdf]

Meta-analysis of the association between SCARB1 polymorphism and fasting blood lipid levels

SUPPLEMENTARY MATERIALS

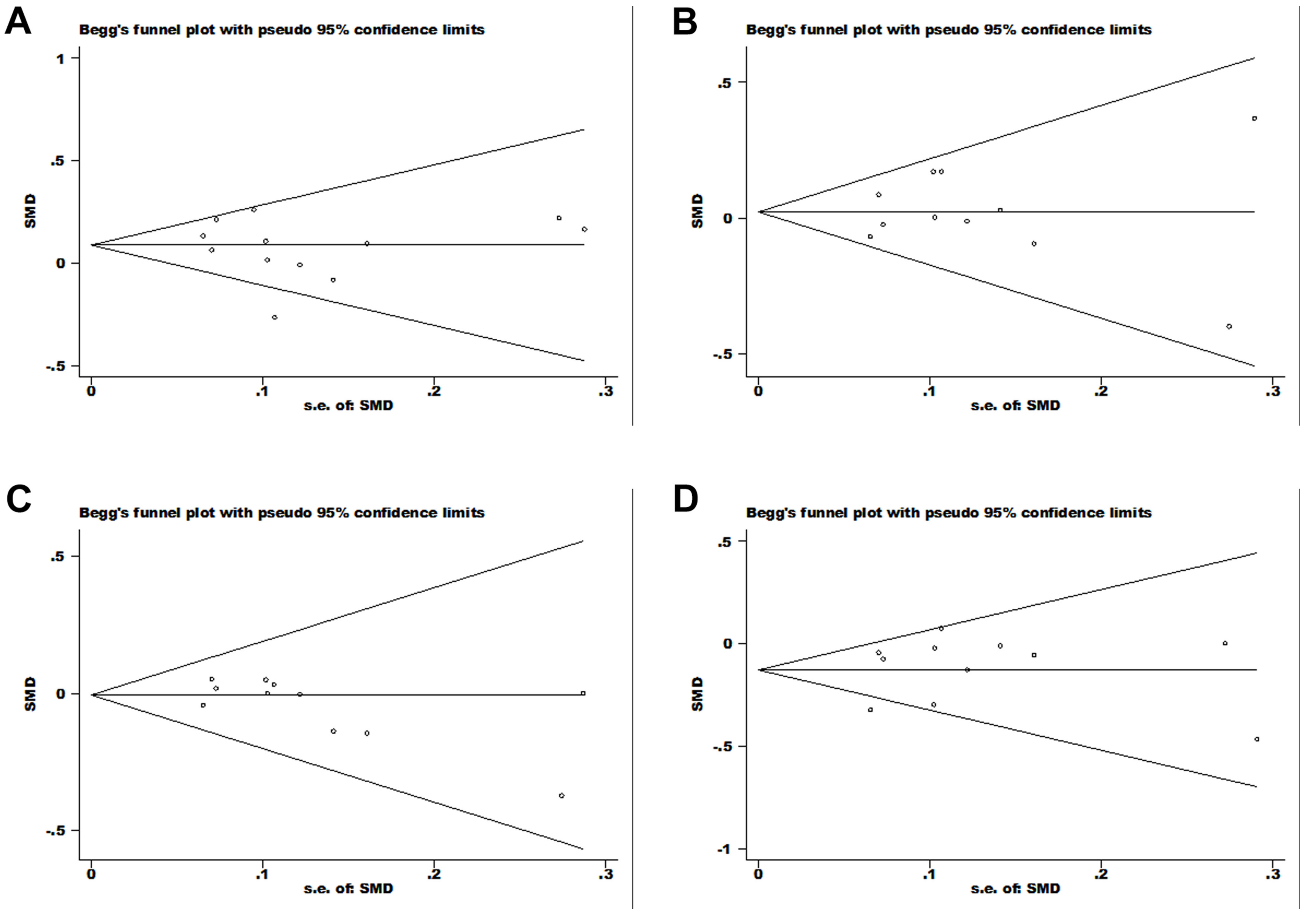

Supplementary Figure 1: Begg's funnel plot of publication bias in the meta-analysis of the association of SCARB1 rs5888 polymorphism with fasting blood lipid levels in the male population. (**A**) HDL-C; (**B**) LDL-C; (**C**) TC; and (**D**) TG.

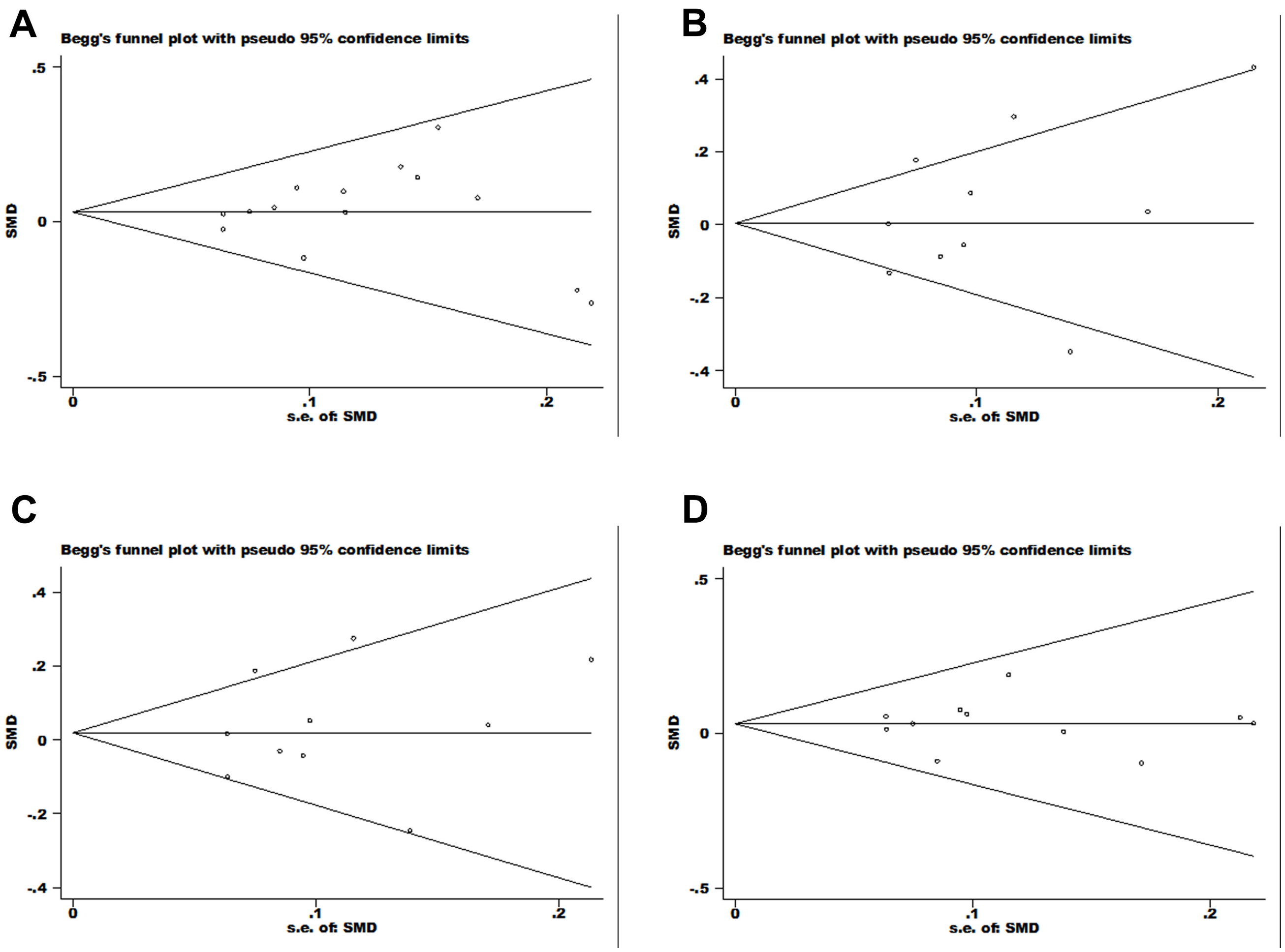

Supplementary Figure 2: Begg's funnel plot of publication bias in the meta-analysis of the association of SCARB1 rs5888 polymorphism with fasting blood lipid levels in the female population. (**A**) HDL-C; (**B**) LDL-C; (**C**) TC; and (**D**) TG.

Supplementary Table 1: PRISMA 2009 checklist. See Supplementary Table 1.

Supplementary Table 2a: Worksheet for assessment of methodologic quality of studies using STREGA guidelines. See Supplementary Table 2a.

Supplementary Table 2b: Completed STREGA checklist for all studies. See Supplementary Table 2b.
